# Healthcare Waste Status and Handling Practices during COVID-19 Pandemic in Tepi General Hospital, Ethiopia

**DOI:** 10.1155/2021/6614565

**Published:** 2021-01-30

**Authors:** Besufekad Mekonnen, Nahom Solomon, Wondimagegn Wondimu

**Affiliations:** ^1^Department of Public Health, College of Medicine and Health Sciences, Mizan-Tepi University, Mizan Aman, Ethiopia; ^2^Department of Epidemiology and Biostatistics, School of Public Health, College of Medicine and Health Sciences, Mizan-Tepi University, Mizan Aman, Ethiopia

## Abstract

**Background:**

Mismanagement of healthcare waste (HCW) during the COVID-19 pandemic can facilitate the transmission of coronavirus. Regarding this problem, there is gap of evidence in Ethiopia, and this study aimed to assess the HCW generation rate and management in Tepi General Hospital, southwest Ethiopia.

**Methods:**

Institution-based cross-sectional and case studies were conducted. The total amount of waste generated and its type among various case teams were compared using the Kruskal–Wallis test. Spearman's rank correlation coefficient (*r*) was used to assess the correlation between the total numbers of patients and the total amount of HCW generated. Qualitative data were transcribed verbatim, translated to English, and analyzed with Open Code version 4.02 software, and content analysis was followed.

**Results:**

The total mean weight (±SD) of waste generation rate in all service units of the hospital was 492.5 ± 11.5 kg/day. The higher proportion (61.9%) of the total HCW produced was general waste and the remaining (38.1%) was hazardous waste. There was a statistically significant (*X*^2^ = 82.1, *p* < 0.001) difference in daily HCW generation rate among different case teams. Similarly, the hospital waste generation amount and total patient flow had a strong positive linear relationship (*r* = 0.7, *p*=0.032). COVID-19-related medical wastes were not properly handled, segregated, stored, and disposed. There was a scarcity of resources needed to manage HCW, and available resources were utilized poorly. Overall, healthcare wastes were managed as usual (pre-COVID-19).

**Conclusion:**

The mean HCW generation rate in Tepi General Hospital was high. Overall, wastes were mismanaged, and COVID-19-related HCWs have been managed as usual. Availing of important resources and training the concerned bodies should be considered during the crisis of COVID-19.

## 1. Introduction

Healthcare waste (HCW) is a collection of waste generated by healthcare establishments, research facilities, laboratories, and home-based healthcare such as dialysis and insulin injection. Broadly, HCW can be classified as hazardous (healthcare risk) and nonhazardous (general) waste. Nonhazardous waste does not pose any specific biological, chemical, radioactive, or physical hazard, whereas hazardous waste can pose several health and environmental risks [1, 2].

Hazardous HCW contains potentially harmful microorganisms that can infect hospital communities and the general public. It may also be a source for drug-resistant microorganisms that spread from health facilities into the environment. In general, health risks associated with HCW and by-products include sharps-inflicted injuries, toxic exposure to pharmaceutical products, chemical burns, air pollution, thermal injuries, and radiation burns [3]. In low-income countries, including Ethiopia, HCW is often not separated into hazardous or nonhazardous waste, making the real quantity of hazardous waste much higher [3, 4]. The current pandemic of coronavirus disease-19 (COVID-19) brings an additional challenge in the waste management of healthcare facilities.

Hospitals produce more waste than usual during COVID-19. The diagnosis and treatment of COVID-19 produce wastes that include masks, gloves, gowns, and other protective equipment that could be infected with the virus. The amount of single-use plastics being produced is also greatly increased [5–7]. Inappropriate management of waste, especially during COVID-19, can predispose healthcare workers, patients, and the general public to coronavirus since the virus can survive on inanimate objects and different surfaces in the hospital [8–10].

The World Health Organization (WHO) recommends that all HCWs produced during the care of COVID-19 patients should be collected safely in designated containers and bags, treated, and then safely disposed of or treated, or both, preferably on-site. Furthermore, if waste is moved off-site, it is critical to understand where and how it will be treated and destroyed. All who handle HCW should wear appropriate personal protective equipment (PPE) and perform hand hygiene after removing it [11]. Similarly, as per the recommendations of both the Ministry of Health (Ethiopia) and the Ethiopian Public Health Institute, all HCWs produced during the care of COVID-19 patients must be considered as infectious waste and should be collected safely in designated containers and bags, treated, and then safely disposed [12].

Ethiopia is already stressed with hospital waste before the COVID-19 pandemic, and currently, there is an unpredicted increase in the volume of medical waste [4]. Confirming that COVID-19-related hospital waste is timely, tidy, efficiently, and harmlessly disposed and end up in the safest dumpsite has also become an important part of the battle against the pandemic. Evidence on the HCW generation rate, type of waste generated, and its management system is very important in designing proper hospital waste management systems during the COVID-19 pandemic. However, in Ethiopia, there is no study performed on the amount and types of waste generated from healthcare facilities during the COVID-19 pandemic. Therefore, this study aimed to assess the HCW generation rate and its management in Tepi General Hospital, Southwest Ethiopia.

## 2. Materials and Methods

### 2.1. Study Area and Period

Institution-based cross-sectional and case studies were conducted in Tepi General Hospital from July 1 to 30, 2020. The hospital is found in Southern Nations, Nationalities, and People's Region State, and it is one of the newly constructed hospitals in Southern Ethiopia. It was established in 2014 and providing healthcare services for more than six hundred thousand populations. Currently, it is serving as COVID-19 isolation and treatment center for people of the Sheka zone and partly for those from the other three zones of Southwest Ethiopia. The facility had 188 beds in four wards which include surgical, medical, pediatric, and gynecology/obstetrics. Outpatient departments (OPD) include adult OPD, pediatrics OPD, maternal and child health clinic, antiretroviral clinic, tuberculosis clinic, emergency case team, follow-up clinic, and others. Moreover, the hospital had a pharmacy, laboratory, and imaging (radiology) case teams. In the first quarter of 2020, the average monthly inpatient and outpatient visits of the hospital were 228 and 3562, respectively.

### 2.2. Sample Size and Sampling Techniques

All service units (case teams) in the hospital (inpatients, outpatients, laboratory, operation theater, pharmacy, radiology, and kitchen) were considered as a source of HCW generation. A purposively selected 42 workers of the hospital have participated in in-depth interviews.

### 2.3. Data Collection Techniques and Quality Management

The waste generation data were collected by using observational checklists and measurement apparatus. The observational checklists were prepared to observe and evaluate how the hospital segregate, collect, transport, treat, and dispose wastes. A standard weighing scale was used to quantify the generation rate of HCW. HCWs were collected and measured daily for seven consecutive days. Empty plastic buckets of standard colors (black color for general waste, brown color for pharmaceutical waste, yellow color for infectious waste, and red color for pathological waste) were distributed daily to different sections of the hospital. Plastic bags with different colors were kept inside the respective buckets. The buckets and plastic bags were labeled to indicate the different categories of the HCWs, the place of generation, date of collection, and sample number. Waste weighing and recording station was arranged in a convenient site within the vicinity of the hospital. Then, the collected wastes in plastic bags were removed every morning, and the weight was measured at 8:00 AM using a weighing scale, capacity range from 15 kg to 25 kg. The measurement was taken three times, and the mean of three measurements was used as the final weight of wastes.

Seven BSc nurses (data collectors) and two public health officers (supervisors) were recruited and trained for data collection. Before data collection, two-day training was given on the purpose of the study, data quality, type of HCWs, the use and calibration of a weighing scale, infection prevention, and control techniques including proper use of PPE. Data collection guideline was developed and used to facilitate the training. Data were recorded daily in a suitable datasheet. Weight scales were arranged and then calibrated every morning. Moreover, daily on-site supervision was made by investigators and supervisors. The pretest was performed before the actual data collection begins, and the daily meeting was arranged to improve data quality. Accordingly, the amendment was made based on the result of the pretest.

To get details of the current status of waste management systems of the hospital, additional qualitative data were collected from workers who have direct and major contact with waste management through in-depth interviews. Workers include waste handlers and team leaders, nurses, midwives, laboratory technicians, medical doctors, hospital manager, and others. An interview tool (guide) was used for conducting a face-to-face interview. The interview tool guides the participants to discuss on the waste segregation practice of the hospital, how the hospital collects and transports waste, how the hospital finally disposes the waste, and overall, how the waste is managed during COVID-19. All interviews were audio-recorded and field notes were taken. Meanwhile, an on-site observation was made to observe the status of the current waste management systems of the hospital. The number of outpatients and inpatient flow data for the study period was taken from the patient registration office. The number of inpatients and outpatients was used to calculate the daily waste generation of the hospital.

### 2.4. Data Processing and Analysis

The data collected through measurement and observation checklist were entered and compiled by using SPSS version 22 computer software packages. The total amount of waste generated and its type among various case teams were compared using the Kruskal–Wallis test as the data distribution was not homogeneous (with unequal variance) even when transformed. Spearman's rank correlation coefficient (*r*_*s*_) was used for testing the existence of any bivariate correlation between the total numbers of patients and the total amount of healthcare waste generated. In addition, daily and annual waste generation rates were described and presented using tables. The annual healthcare waste generation rate was estimated by multiplying the mean healthcare waste generation rate in kg per day by 365 days (the assumption was each patient who visited the hospital may generate the same amount of HCW throughout the year). Qualitative data were transcribed verbatim, translated to English, and analyzed with Open Code version 4.02 software, and content analysis was followed. Codes and categories were developed, and findings were summarized by supporting with quotes from respective study participants.

### 2.5. Ethical Approval and Consent to Participate

Ethical clearance was obtained from the Ethical Review Committee of Mizan-Tepi University, College of Health Science. Written consent was obtained from each study participant. Each study participant was briefed with the objective, benefit, and procedural steps of the study. Voluntary participation was assured, and the right to interrupt from the interview was clearly stated. Anonymity and confidentiality of the data were also ensured.

## 3. Results

### 3.1. The Waste Generation Rate of the Hospital

A total of 752 patients have visited Tepi General Hospital during data collection time (within one week) in all health service delivery units. Of these, 212 (28.2%) patients were admitted to the inpatient department and 540 (71.8%) were seen at OPDs. The higher proportion (61.9%) of the total HCW produced was general waste and the remaining 38.1% was hazardous or risk waste. The types of hazardous wastes generated in Tepi General Hospital were sharps, infectious, pharmaceutical, and pathological (placenta and blood) wastes. The mean (±SD) generation rate of sharps, infectious, pharmaceutical, and pathological wastes in the studied hospital was 7.2 ± 0.9 (1.5%), 158.7 ± 0.4 (32.2%), 19.2 ± 0.5 (3.9%), and 2.3 ± 0.2 (0.5%) kg/day, respectively. More than a half (0.6) kg of waste was produced by one patient daily. Totally, an estimated yearly waste generation rate of the hospital was 179,762.5 kg/year (Table 1).

### 3.2. Distribution of Hazardous Wastes in Different Service Units

The sharp, infectious, and pharmaceutical hazardous wastes were mainly generated from wards. However, the highest share of the pathological waste was from the gynecology unit. There was no hazardous waste generated from the kitchen. Generally, the maximum and minimum amounts of hazardous wastes were generated from wards and radiology room, respectively (Table 2).

### 3.3. Comparison of Hospital Waste Generation among Different Service Units

The estimated annual total waste generation rate was 179,762.5 kg/year or 956.2 kg/bed/year. The estimated annual healthcare waste generation rate in kg/bed/year for infectious, pathological, pharmaceutical, sharp, and general wastes was 308.1, 4.5, 37.3, 14, and 592.3, respectively.

The highest amount ((48.2%) 237.3 kg/day) of the hospital waste was generated from the kitchen room, whereas that of the lowest amount ((0.9%) 4.3 kg/day) was generated in the radiology room. The Kruskal–Wallis test indicated a statistically significant (*X*^2^ = 82.1, *p* < 0.001) difference in daily healthcare waste generation rate among different case teams. This implies that the type or specialty of case teams was a factor for the generation rate of HCWs. Spearman's rank correlation coefficient (*r*_*s*_) estimation showed a strong positive linear relationship between the amount of hospital waste generation and the total patient flow (*r* = 0.7, *p*=0.032) (Table 3).

### 3.4. Hospital Waste Management Practices

A total of 42 health workers have participated in an in-depth interview (Table 4) and discussed what the waste management practices of the hospital look like. The response of participants and the observational findings are summarized under a few categories.

#### 3.4.1. Category I: Waste Segregation

As we have observed and study participants reported, hospital wastes were temporarily stored using plastic buckets with cover for nonsharp wastes. Safety boxes were used for sharp waste in the emergency room and surgical and gynecological wards. It was also reported that the segregation of HCW by its type at the point of generation and pretreatment of infectious wastes were poorly practiced in the hospital. Majorities of study participants have pointed out that color-coded waste segregation materials were not adequately available in each unit, and front-line health service workers did not segregate those wastes as hazardous and nonhazardous. One of the study participants explained the issue as follows.

There are mixed wastes on the color-coded bins which are due to inappropriate waste segregation practice by concerned bodies of the hospital. (a nurse study participant)

#### 3.4.2. Category 2: Waste Collection and Transport

Almost all study participants have discussed that, though waste from the outpatient department is collected within 24 hours' intervals, the hospital has no fixed time to transport wastes into the designated area. All waste handlers have reported that they have duty gloves but they do not use during waste collection and transportation. However, they have revealed that they lack safety boots and face masks, and for that reason, they are obliged to wear their habitual personal shoes during their routine working time. One of them depicted the concern as follows.

Of course we have gloves but we usually miss to use it. On the other hand, although we need a face mask we could not have that and again we have also a shoe problem, we use our own shoes during cleaning the working area, no boot at all. (a waste handler study participant)

It was observed and study participants reported that the infectious medical HCWs including face masks and nonrisk HCWs are not collected on separate plastic barrel containers; rather, infectious and noninfectious wastes are usually mixed and transported into an incinerator room. However, sharp materials are usually separated and transported with a safety box, while plastics, paper towels, and face masks were transported with large bins made up of plastic material. The following statement from one of the study participants agrees with this.

We give attention to sharp materials and for that, we regularly collect it under separate safety boxes and dispose properly. Sometimes other wastes may be collected together without segregating based on their types. (a nurse study participant)

#### 3.4.3. Category 3: Waste Treatment and Disposal

We found that the waste disposal methods employed by Tepi General Hospital were incineration and the open container that was picked by waste collector firms and plastic bottle houses and end up in the open dumpsite. Study participants have reported that the waste incinerator is used for disposal of all medical infectious wastes such as sharp materials (syringe, needles, blades, and others), anatomical wastes, used face masks, and paper towels. On the other hand, the majority of study participants discussed that wastes including used batteries, broken thermometers, radioactive waste, and PVC plastic-like IV bags were not separated and disposed properly in the vicinity. Again, silver and X-ray films from radiotherapy were not managed and recycled properly. The following are part of the responses from study participants.

Even during COVID-19, the waste handling and disposal habit is not improved and there are many things which need a rapid solution. (a laboratory technician study participant)

Actually we have an incinerator and other waste management systems but I can't say we are fully practicing as recommended and expected of us. We are working for improvements to make our working environment safe. (a medical doctor study participant)

Most study participants have pointed out that COVID-19-related healthcare waste disposal was not arranged by specifically trained staff and special vehicles or supporting materials. They have also mentioned that all healthcare workers and waste handlers did not take training on medical waste management, especially on waste segregation at the time of the COVID-19 pandemic. Standard operating procedures were not established, and the hospital did not have a healthcare waste management committee too.

## 4. Discussion

Medical activities generate waste that should always be discarded at the point of use by the person who used the item. The quantity of HCW generated should always be minimized, and precautions must be taken during their handling particularly in the time of COVID-19 [6, 13]. The average daily HCW generation rate of Tepi General Hospital was 1.88 kg/bed/day, and this is higher than the findings of the studies conducted in Mizan-Tepi University Teaching Hospital and Adama Referral Hospital, Ethiopia, which reported average waste generation rates of 0.164 kg/bed/day and 1.23 kg/bed/day, respectively [14, 15]. The highest waste generation rate in the current study might be due to the COVID-19 pandemic which brings a new threat to the environment especially in low-income countries [16]. The patient flow was positively correlated to the waste generation rate in the studied hospital. The pandemic of the COVID-19 brings an increased patient flow to the hospital which in turn leads to a high amount of wastes generated, and therefore, proper waste management practices should be implemented.

The hazardous HCW is associated with a risk of infecting workers handling HCW and others. Consequently, it requires careful waste management compared to general waste. This study revealed that 32.37% of HCWs in Tepi General Hospital were hazardous. This is lowest compared to other findings from studies conducted in Ethiopian hospitals [14, 17]. The discrepancy might be due to the differences in hospital size, patient flow, and waste management practices among different hospitals. However, our finding is two times higher than the WHO report which states that 15% of HCWs are hazardous [2].

The higher proportion of hazardous waste generation may be attributable to poor segregation practice in the studied hospital. As it was noted from this study, the resources required to segregate wastes were inadequate, and the front-line health service workers did not segregate wastes using available materials. During the pandemic of COVID-19, the priority attention was given mainly to sharp materials by ignoring different infectious wastes such as facemasks. However, facemasks might be the source of infection for coronavirus. As a result, hazardous wastes should be segregated properly. Proper segregation of waste can reduce the treatment and disposal costs and the risks of infecting workers handling HCW. Moreover, the part of the HCW that is hazardous and requires special treatment could be reduced to some 2–5% if the hazardous part was immediately separated from the other wastes [9, 13]. Therefore, the poor segregation practice in the studied hospital needs important attention especially during the crisis of COVID-19.

The waste collection, transport, treatment, and disposal practices of the Tepi General Hospital were substandard. In this crisis time, all people in the country are recommended to protect themselves from COVID-19 by using different PPE. Surprisingly, the waste handlers, the priority groups to use PPE, in Tepi General Hospital were facing the scarcity of the PPE such as safety boots and facemasks. They were not using their duty gloves, and this might be due to the lack of awareness creation strategies such as training since they were also reporting that they did not take any training which guides them on how to handle wastes during COVID-19. Wastes were also collected with an open container, and some of the wastes were ended up with open dumping. Furthermore, the absence of standard operating procedures and the healthcare waste management committee was an important finding in the hospital. This is in contrary to the national and international (WHO) recommendations of proper waste management during COVID-19 and the experiences of other countries [11, 12, 18–21].

## 5. Conclusion

The mean HCW generation rate in Tepi General Hospital was high, and overall wastes were mismanaged. Although available resources were poorly utilized, the scarcity of vital inputs for proper waste management was one of the important findings. Moreover, COVID-19-related HCW was being managed as usual, and no different waste management method was employed so far. Availing important resources and training the concerned bodies should be considered during the crisis of COVID-19.

## 6. Limitations of the Study

Due to the lack of comparable studies conducted during COVID-19, the comparison was performed with studies that were performed before the pandemic. Moreover, our study was conducted in single site and it could not be generalizable to other settings. Thus, large-scale studies which can also identify the potential determinants of COVID-19-related waste generation and management practices are recommended.

## Figures and Tables

**Table 1:** The amount of HCW generation rate and types of waste in Tepi General Hospital, Ethiopia.

Types of wastes	Weight of daily generated waste (mean in kg ±SD)	%	Weight of daily generated waste (kg/patient ^*∗*^/day)	Weight of daily generated waste (kg/bed ^*∗∗*^/day)	Weight of yearly generated waste (kg/year)
General waste	305.1 ± 2.1	61.9	0.4057	1.6228	111,361.5
Sharps waste	7.2 ± 0.9	1.5	0.0096	0.0383	2628
Pharmaceutical waste^#^	19.2 ± 0.5	3.9	0.0255	0.1021	7008
Infectious waste	158.7 ± 0.4	32.2	0.2110	0.8441	57,925.5
Pathological waste	2.3 ± 0.2	0.5	0.0030	0.0122	839.5
Total	492.5 ± 11.5	100	0.6548	2.6195	179,762.5

^*∗*^752 total patients visited the hospital during the data collection period;  ^*∗∗*^there were a total of 188 beds in the hospital during the data collection time; ^#^nonhazardous pharmaceutical wastes were included in general waste.

**Table 2:** Distribution of types and amount of daily hazardous waste generation rate at each service unit of Tepi General Hospital.

Name of case teams	Sharps (kg/day)	Infectious (kg/day)	Pharmaceutical (kg/day)	Pathological (kg/day)	Total hazardous waste (kg/day)
Wards	2.4	65	8	0	75.4
Gynecology	1.5	31	4.1	1.8	38.4
Laboratory	1.3	30	5	0.3	36.6
OPD	1.7	29.7	2	0.2	33.6
Radiology	0.3	3	0.1	0	3.4
Kitchen	0	0	0	0	0

**Table 3:** Comparison of patient flow, total HCW generation, and its type using the Kruskal–Wallis test in each case team in Tepi General Hospital, Ethiopia.

Types of case teams	Mean value (kg/day)			
	Patient flow, *n* (%)	Total hospital wastes, *n* (%)	General HCW, *n* (%)	Hazardous HCW, *n* (%)
Wards	126 (16.7)	83.1 (16.9)	7.7 (2.5)	75.4 (40.2)
Gynecology	91 (12.1)	48.9 (9.9)	10.5 (3.4)	38.4 (20.5)
Laboratory	154 (20.5)	44.6 (9)	8 (2.6)	36.6 (19.5)
OPD	297 (39.5)	74.3 (15.1)	40.7 (13.3)	33.6 (17.9)
Radiology	84 (11.2)	4.3 (0.9)	0.9 (0.3)	3.4 (1.8)
Kitchen	0 (0)	237.3 (48.2)	237.3 (77.8)	0 (0)
*X* ^2^	125.3	82.1	60.6	28.2
Degree of freedom	5	9	10	9
*p* value	0.002	0.001	0.004	0.001

**Table 4:** In-depth interview participants for describing waste management practices of Tepi Hospital, 2020.

Hospital workers	Number
Environmental health expert	1
Infection prevention team leader	1
Department heads	7
Waste handler team leaders	3
HCW handlers	7
Nurses	7
Midwives	3
Pharmacists	3
Laboratory technicians	3
Medical doctor	4
Radiology technician	1
Medical director	1
Hospital manager	1

## Data Availability

The data that support the findings of this study can be obtained from the corresponding author upon reasonable request.

## Abbreviations

COVID-19: Coronavirus disease 2019
HCW(s): Healthcare waste(s)
kg: Kilogram
OPD: Outpatient department
PPE: Personal protective equipment
SD: Standard deviation
SPSS: Statistical Package for Social Sciences
WHO: World Health Organization.
